# Heightened metabolic responses in NK cells from patients with neuroblastoma suggests increased potential for immunotherapy

**DOI:** 10.3389/fonc.2022.1004871

**Published:** 2022-10-07

**Authors:** Karen Slattery, Megan Breheny, Elena Woods, Sinead Keating, Kiva Brennan, Caroline Rooney, Sindhu Augustine, Aishling Ryan, Cormac Owens, Clair M. Gardiner

**Affiliations:** ^1^ School of Biochemistry and Immunology, Trinity Biomedical Sciences Institute, Trinity College, Dublin, Ireland; ^2^ Department of Oncology, Children’s Health Ireland at Crumlin, Dublin, Ireland

**Keywords:** neuroblastoma, natural killer (NK), metabolism, immunotherapy, circulating, cancer, pediatric, mitochondria

## Abstract

High risk neuroblastoma is responsible for 15% of deaths in pediatric cancer patients. The introduction of anti-GD2 immunotherapy has significantly improved outcomes but there is still only approximately a 50% 5 year event-free-survival for these children and improvements in treatments are urgently required. Anti-GD2 immunotherapy uses the patients’ own immune system to kill cancer cells. In particular, Natural Killer (NK) cells kill antibody coated tumor cells by a process called antibody dependent cellular cytotoxicity (ADCC). However, our previous work has highlighted metabolic exhaustion of NK cells in circulating blood of adult cancer patients, identifying this as a potential therapeutic target. In this study, we investigated circulating NK cells in patients newly diagnosed with neuroblastoma. We found evidence of activation of NK cells *in vivo* by the cancer itself. While some evidence of NK cell dysfunction was observed in terms of IFNγ production, most results indicated that the NK cell compartment remained relatively intact. In fact, some aspects of metabolic and functional activities were actually increased in patients compared to controls. Glycolytic responses, which we show are crucial for ADCC, were actually enhanced in patients and CD16, the NK cell receptor that mediates ADCC, was also expressed at high levels in some patients. Overall, the data suggest that patient NK cells could be harvested at diagnosis for subsequent beneficial autologous use during immunotherapy. Enhancing glycolytic capacity of cell therapies could also be a strategic goal of future cell therapies for patients with neuroblastoma and indeed other cancers.

## Introduction

Neuroblastoma is the most common extracranial tumor in children and accounts for approximately 15% of childhood cancer deaths (1, 2). It is classified into low, medium and high risk neuroblastoma based on various clinical and molecular criteria, and while the former groups respond well to treatment, high-risk neuroblastoma remains a significant clinical challenge (3, 4). The introduction of anti-GD2 antibody immunotherapy in 2010, which preferentially identifies tumor cells for immune killing, was the most recent major breakthrough and increased 2 year event free survival (EFS) from 46% to 66% for this cohort (5), with a five year EFS of 56.6% most recently reported (5, 6). Despite this impressive improvement, the figures remain bleak and there is an urgent need to develop new treatment options for these patients. Unfortunately, most new or improved cancer therapies are developed for adult cancers, in part because they are more common and have an obvious commercial potential for pharmaceutical companies (7). Protocols for children are generally adapted from the adult situation despite the fact that pediatric cancers are often very different in terms of molecular origins, mutational burden, and are usually associated with cell developmental or differentiation origins rather than lifestyle factors (2, 8). This is particularly relevant in neuroblastoma as it is a cancer uniquely found in children. Furthermore, in an era of cancer immunotherapy successes, it is important to develop and tailor specific immunotherapies for the target audience. While neuroblastoma has already demonstrated the potential to respond to immunotherapy, future impact is likely to come from an understanding of pediatric immunology and how to optimally deploy immunotherapy in this vulnerable cohort.

Natural Killer (NK) cells are immune cells that can kill cancer cells (9). In addition to preventing cancer by immunosurveillance, autologous NK cells are used therapeutically in a number of different cancers including high risk neuroblastoma. In addition to direct cytotoxicity, NK cells kill therapeutically by a process termed antibody dependent cellular cytotoxicity (ADCC) (10). Here, NK cells recognize anti-GD2 coated neuroblastoma cells and kill them using a suite of cytotoxic molecules including granzyme B and perforin (11). In recent years, it has become clear that cellular metabolism is deeply interlinked with immune cell function (12). Particular immune cells adopt particular metabolic configurations that support their specialized activities. Ourselves and others have shown that while resting NK cells tend to use oxidative phosphorylation (oxphos) for glucose metabolism, both glycolysis and oxphos metabolic pathways are upregulated in NK cells in response to cytokine stimulation (13). These metabolic changes are important for optimized effector functions of NK cells.

It is also known that while the immune system, and particularly NK, cells are important in protecting us from cancer, immune cell functions become impaired as cancer develops (14, 15). NK cell exhaustion has been reported in many cancer types and development of specific NK cell checkpoint inhibitors is an active field of research (16–18). This exhaustion is a barrier to optimal deployment of NK cell immunotherapy. We have previously reported impaired NK cell functional defects in a cohort of women with metastatic breast cancer (19). Circulating NK cells from patients were characterized by severe metabolic dysregulation with many mitochondrial changes including high mitochondrial mass, high levels of mitochondrial ROS (mtROS), punctate fissed mitochondrial organization and an inability to engage in oxphos in response to stimulation. This largely older cohort of metastatic patients are on the severe end of the cancer spectrum and such a profound phenotype was not surprising. Indeed, even healthy adults have accumulated cellular damage through a lifetime of experiences (20, 21). Given the potential impact of aging, co-morbidities and immunological exposures, we hypothesized that ‘younger’ NK cells from pediatric cancer patients might be metabolically more plastic than those from adults and that differences might be informative in terms of tailoring immunotherapy for pediatric patients. We therefore undertook an investigation into the metabolic fitness of circulating NK cells in children recently diagnosed with neuroblastoma prior to any medical intervention.

## Materials and methods

### Subjects and ethics

Blood samples were obtained from normal healthy adult donors (age and sex not recorded for ethical reasons), healthy pediatric donors (undergoing elective surgical procedures, n=9) and treatment-naïve, newly diagnosed neuroblastoma patients (n=6). Sex, average age and age ranges are in **Table 1**. Blood was collected in EDTA coated tubes. The Trinity College Faculty of Science, Technology, Engineering and Maths Research Ethics Committee provided ethics for analysis of healthy adult donor blood. All healthy adult donors for this study provided written consent. The ethics committee of Children’s Health Ireland at Crumlin (formerly Our Lady’s Children’s Hospital) approved the study on healthy pediatric donors and neuroblastoma patients, and parents/guardians provided written consent.

**Table 1:** Summary of patients and participants.

	Neuroblastoma	Pediatric controls
Numbers	n=6	n=9
Female/male	4/2	7/2
Average age (years)	3.1	7.7
Age range (years)	1.2-15	3-15

### Cell culture

Peripheral blood mononuclear cells (PBMC) were isolated by Lymphoprep (Axis-Shield) gradient on the same day blood sample was drawn, and experiments were carried out on the freshly isolated PBMC. For seahorse analysis and confocal microscopy, NK cells were purified using either the MoJoSort human NK cell isolation kit (BioLegend) or the EasySep Human NK Cell Isolation Negative Selection Kit (StemCell) according to the manufacturer’s instructions. NK cells were routinely 85-95% pure. Unless stated otherwise, 5×10^6^ cells/mL PBMC or purified NK cells were incubated at 37°C for 18 hours in RPMI 1640 GlutaMAX medium (Life Technologies/Invitrogen) supplemented with 10% fetal calf serum (FCS), 1% penicillin/streptomycin (Invitrogen). Cells were stimulated with interleukin (IL)2 (500 IU/mL; National Cancer Institute) or IL12 (30 ng/mL; Miltenyi Biotec) and IL15 (100 ng/mL; National Cancer Institute). Where indicated, cells were treated with 2DG (2.5mM, Sigma), oligomycin (40nM, Sigma), isotype control (IgG2a, κ, 2.5μg/ml) or anti-GD2 antibody (Clone 14.G2a, 2.5μg/ml).

### Flow cytometry analysis

Cells were stained surface for 20 min at 4°C with saturating concentrations of titered Abs, fixed and permeabilized and then stained intracellularly for 20 min at 4°C with saturating concentrations of titered Abs: CD56 (HCD56/NCAM16.2), CD3 (SK7/UCHT1), granzyme B (GB11), IFNγ (B27), CD71 (M-A172), CD69 (L78), CD98 (UM7F8), S6 ribosomal protein phosphorylated on serine 235/6 (pS6), and eukaryotic translation initiation factor 4E-binding protein 1 (4E-BP1) phosphorylated on Thr37/46 (236B4, Cell Signalling Technology). A viability dye was included in every panel (LIVE/DEAD Near-IR, Bio Sciences). The gating strategy is shown in **Supplementary Figure 1A** and a full list of antibodies in **Supplementary Figure 1B**. Samples were analyzed on a BD Canto or BD Fortessa flow cytometer.

### Mitochondrial flow cytometry analysis

Mitochondrial membrane potential (MMP) was measured by staining cells for 20 min with tetramethylrhodamine methyl ester (TMRM, 100 nM—Thermo Fisher), oligomycin (2 µM) and carbonyl cyanide p-trifluoro-methoxyphenyl hydrazone (FCCP, 2 µM) were used as positive and negative controls, respectively (**Supplementary Figure 1C**). Mitochondrial mass was measured *via* staining of cells for 20 min MitoTracker Green (100nM—Thermo Fisher). Adenosine triphosphate (ATP) synthase analysis was performed by detecting the expression of the ATP5B subunit of the (3D5, Abcam) *via* intracellular flow cytometry staining. Mitochondrial superoxide levels were measured *via* staining of cells for 15 min with MitoSOX (Thermo Fisher). Rotenone (20 µM) was used as a positive control (**Supplementary Figure 1D**).

### Kynurenine uptake assay

PBMC were surfaced stained with NK cell markers. Cells were washed and resuspended in 100ul Kynurenine (200μM, made up in HBSS). Leucine (5mM) and BCH (10mM) were added as negative controls (**Supplementary Figure 1E**). Tubes were made up to 400ul with HBSS and put in a water bath at 37°C for 4 minutes. 4% PFA was used to fix cells at room temperature for 15 minutes in the dark. Cells were then washed twice with FACS buffer and analyzed *via* flow cytometry.

### Seahorse analysis

Determination of oxygen consumption rate (OCR) representing oxphos or extracellular acidification rate (ECAR) indicating glycolysis was detected by XFp extracellular flux analyzer (Agilent Technologies). NK cells were stimulated for 18 hours with IL2. In order to adhere NK cells to the bottom of the seahorse plate, cell plates were coated with Cell-Tak (6 µg/mL). The Cell-Tak was diluted in sodium bicarbonate (0.1M) with 0.15% (v/v) NaOH (1M) and added to the bottom of each well (25 µl). It was left at room temperature (RT) for a minimum of 20 min, removed from the plate and each well was washed two times with sterile ddH2O. NK cells were washed two times in GlutaMAX seahorse media supplemented with glucose (1M), adjusted to pH 7.4. NK cells were added to each well (2.5×105 cells, 180 µl), while seahorse media were used in the blank wells. The cell plate was centrifuged at 200g for 3 min with no brake, and then placed in a non-CO2 incubator for 30 min prior to metabolic analysis. During the assay, the following inhibitors were added in order—oligomycin (2 µM), FCCP (0.5 µM), rotenone (100 nM)+antimycin A (4 µM) and 2-DG (30 mM).

### Confocal imaging of mitochondrial morphology

Purified NK cells (8×10^5^ cells and >90% purity) were stained using Mitospy CMX Ros (250 nM, Biolegend) for 30 min at 37°C and fixed in 2% paraformaldehyde (PFA, Sigma) for 15 min at RT, prior to nuclear staining with 4’,6-diamidino-2-phenylindole, dihydrochloride (DAPI, 300 nM, Thermo Fischer Scientific) for 5 min at room temperature. NK cells were mounted using Mowiol (Sigma) and imaged on a Leica SP8 inverted motorized microscope equipped with a ×63/1.4 N.A. oil objective and 405 nm diode and Leica white laser lines. Z-stacks at 0.2 µm increments were captured using an HyD detector in conjunction with Leica LAS X acquisition software.

### ADCC and degranulation assays

Kelly target cells represent high-risk neuroblastoma cells which express high levels of the GD2 antigen. Kelly cells were washed thoroughly out of media twice using warm PBS at 300g for 4 minutes. The cells were counted and resuspended at 3x10^6^cells/ml + 20μM Calcein AM dye and incubated at 37°C for 30 minutes. Labelled Kelly cells were thoroughly washed in wash buffer (20%FCS in PBS) 3 times. The cells were resuspended at 0.3x106 cells/ml and allowed to rest for 1 hour at 37°C.

PBMC were counted, washed and plated in a 96 well plates according to the E:T ratios in triplicate (10:1, 5:1 and 1:1). Kelly cells were coated with either isotype control (IgG2a, κ) or anti-GD2 antibody (Clone 14.G2a, 2.5μg/ml). Kelly cells were seeded into a 96 well plate with PBMC. Kelly cells were also plated on their own without PBMC, to measure spontaneous release of Calcein AM. Additionally, Kelly cells alone were plated in the presence of 8ul of 10% TritonX to measure maximum release of Calcein AM. Cells were incubated for 4 hours at 37°C. For metabolic analyses, oligomycin (40nM) or 2DG (2.5mM) was added for the duration of the incubation.

To incorporate the degranulation assay, Golgi stop (BD Pharmingen, 1/400) and anti-CD107a (LAMP1, 1.2ul) is added to the wells.

At the end of the assay, cells were spun down and 75μl of supernatant was aspirated and transferred to a black 96 well plate. The fluorescence of Calcein AM in the supernatant was measured using the following setting of a Spectra Max spectrophotometer: Excitation: 485nm, Auto cut off-10nm, Emission: 525nm, Plate type: Costar 96well plate black, Flashes: 6, Gain: Auto. Triplicate values were averaged and the percentage killing was calculated using the following equation:


$$\frac{\left(Fluorescence\;reading–Spon\tan eous\;release\right)\times 100}{\left(Maximum\;release-Spon\tan eous\;release\right)}$$

When continuing with the degranulation assay, cells of the highest ratio were stained for NK cell surface markers and fixed, and analyzed by flow cytometry.

### Statistical analysis

All data was analyzed using GraphPad Prism 8 software. Data was determined to be parametric or not using the D’Agostino-Pearson normality test. Data was then analyzed using the student-t test when two data sets were being compared, or the one/two-way ANOVA test when more than two data sets were being compared. If no statistic is shown, the results were non-significant.

## Results

### NK cells in patients with neuroblastoma have an activated phenotype *in vivo*

**Figure 1:**
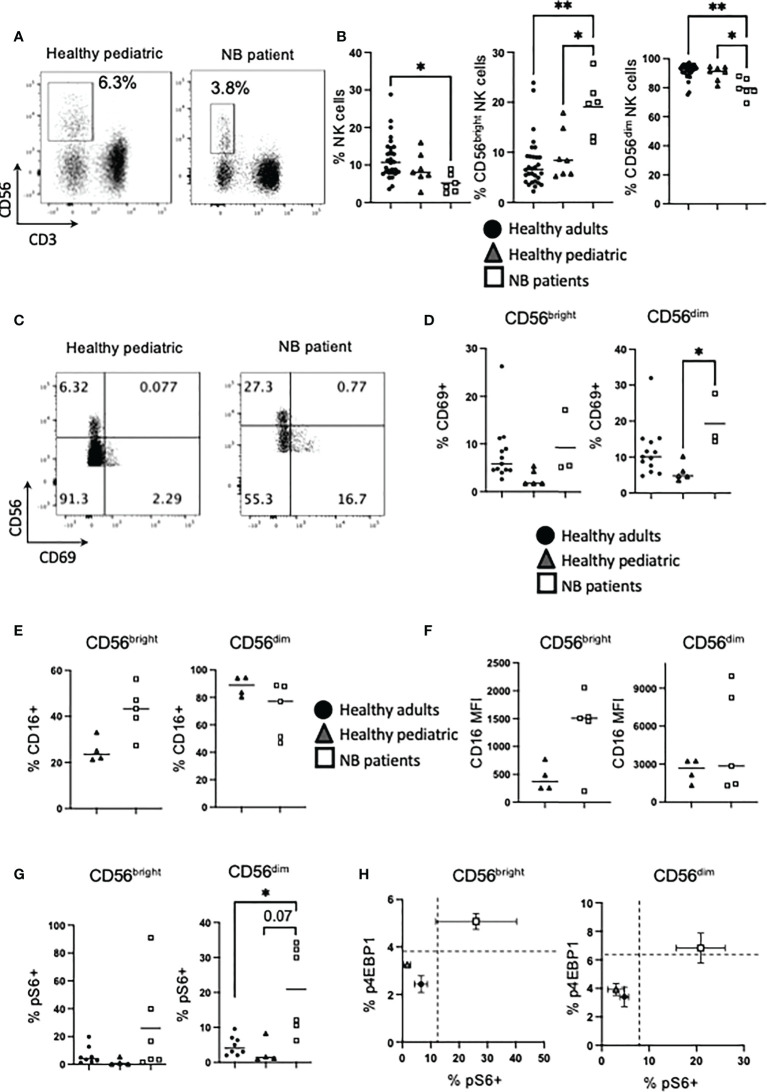
NK cells from NB patients are activated and express high levels of CD16. PBMC were isolated from fresh blood of healthy adult donors, healthy pediatric donors and NB patients. **(A, B)** PBMC were stained for CD56 and CD3 directly ex vivo and analyzed by flow cytometry to identify the frequency of NK cells (CD56+ CD3−) and the CD56^bright^ and CD56^dim^ subsets. **(C–H)** PBMC were stained for CD69, CD16 and pS6 analyzed by flow cytometry. Dots represent individual donors and horizontal bars show the mean. **(H)** Dots represent the mean and error bars show the SEM. N=3–40. Samples were compared using the Two-way ANOVA test or Student’s t-test, *p<0.05, **p<0.01.

Patients with neuroblastoma had a significantly lower frequency of NK cells compared to healthy adult controls (**Figure 1**). In our study, we also included a pediatric control group to challenge the current immunotherapy development pathways that assume pediatric and adult immune systems are equivalent. Healthy pediatric controls had an average NK cell frequency between the two other comparator groups, and there was no statistically significant difference between neuroblastoma patients and pediatric controls (**Figures 1A, B**).

At its simplest, circulating NK cells are characterized by two main subsets. These are reviewed in depth elsewhere but in brief, CD56^dim^ cells account for approximately 90% of peripheral NK cells, they usually co-express CD16 and constitutively express cytotoxic molecules for killing target cells. In contrast, the minor subset of CD56^bright^ cells, expresses CD56 at higher levels and produces large amounts of IFNγ upon cytokine activation (22, 23). Analysis of these functional subsets showed a skewed distribution with a significantly higher frequency of CD56^bright^ NK cells (and corresponding decrease in CD56^dim^ cells) in patients with neuroblastoma (median of 6.6%, 8.5% and 20% for CD56^bright^ NK cells from healthy adults, healthy children and NB patients respectively), compared with either control cohort (**Figure 1B**). Using CD69 as a marker of activation in circulating NK cells, CD56^dim^ cells in neuroblastoma patients were more activated, reflecting *in vivo* activation by the cancer milieu (**Figures 1C, D**). Given that CD16 is a key receptor for mediating therapeutic ADCC, we measured CD16 expression on NK cells and found that while healthy pediatric controls had roughly similar patterns of expression to that found on adult NK cells (22), unstimulated NK cells from patients with neuroblastoma had a skewed pattern with a much higher frequency of CD16 co-expression on CD56^bright^ cells (**Figure 1E**). Intriguingly, the data also demonstrated that NK cells from four of 5 patients co-expressed CD16 receptor at very high levels on CD56^bright^ cells, while two of these 5 patients also expressed CD16 at high levels on CD56^dim^ cells compared to pediatric controls (**Figure 1F** and **Supplementary Figure 2**). Given that there was some evidence of *in vivo* activation of NK cells in patients with neuroblastoma, we investigated for increased cell signalling, focusing on mTORC1 as it is a key regulator of NK cell function and metabolism. A higher frequency of CD56^dim^ cells from neuroblastoma patients were positive for phosphorylated ribosomal protein 6 (pS6), a readout of mTORC1 activity, *ex vivo* (**Figure 1G**). The frequency of NK cells positive for phosphorylated 4EBP1, an alternative mTORC1 target, were lower but followed similar trends (**Supplementary Figure 3**). Indeed NK cells from healthy adults and children clustered together, and separate from children with neuroblastoma, when both mTORC1 readouts were plotted against each other (**Figure 1H**). Thus, there is a higher frequency of CD56^bright^ NK cells in patients with neuroblastoma compared with control groups with some patients having higher frequency and expression levels of CD16, which is required for ADCC. Furthermore, circulating NK cells in the patients showed evidence of cell activation *in vivo.*

### NK cells from patients with neuroblastoma have a specific defect in IFNγ production but enhanced granzyme B expression

**Figure 2:**
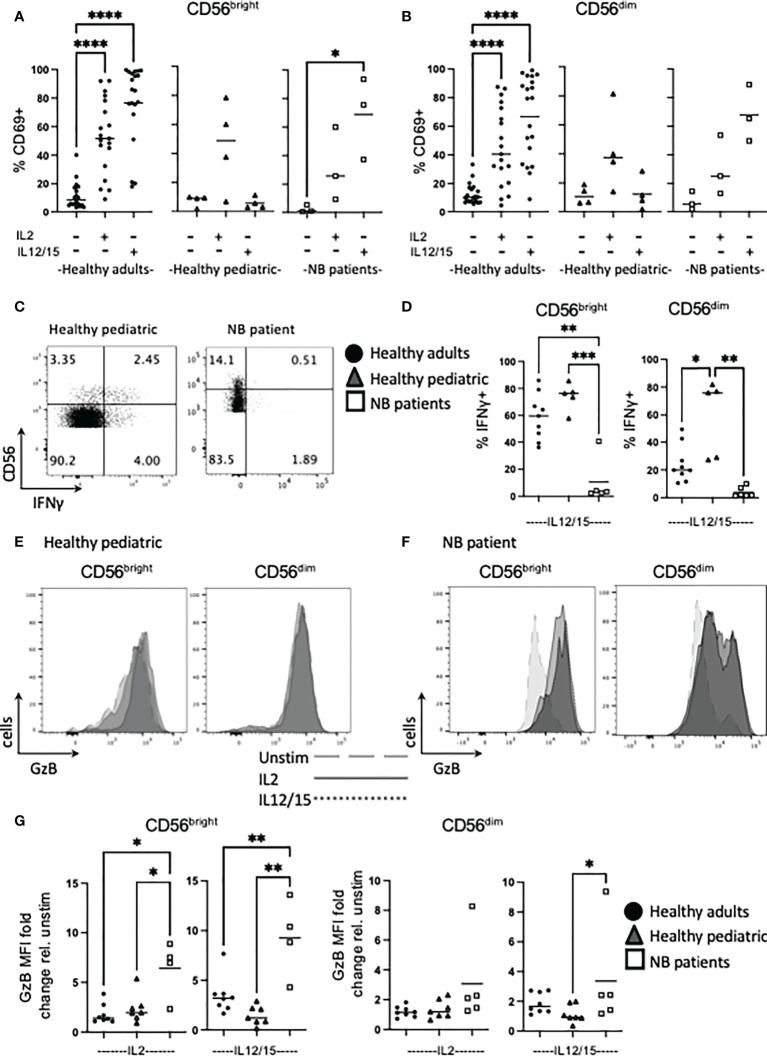
Cytokines induce high levels of Granzyme B in NK cells from NB patients. PBMC were isolated from fresh blood of healthy adult donors, healthy pediatric donors and NB patients. Cells were stimulated with IL2 (500 IU/mL) or IL12 (30 ng/mL) and IL15 (100 ng/mL) at 37°C for 18 hours where indicated. **(A–G)** Cells were stained for CD69, IFNγ and granzyme B (GzB) and analyzed by flow cytometry. **(C, E, F)** Figures are representative of pooled data. Dots represent individual donors and horizontal bars show the mean, N=3-20. Samples were compared using the Two-way ANOVA test, *p<0.05, **p<0.01, ***p<0.001, ****p<0.0001.

To maintain immune homeostasis and prevent detrimental immune responses, immune cells require signals for activation. Therefore, the ability of NK cells to respond to cytokines to engage functional responses was investigated. While only a low frequency of unstimulated NK cells from healthy adults expressed the early activation antigen CD69, this increased significantly in response to IL2 overnight, and even more in response to IL12/15 stimulation for both CD56^bright^ and CD56^dim^ subsets, as expected (**Figures 2A, B**). Healthy pediatric control NK cells upregulated CD69 in response to IL2 but not IL12/15. In contrast, circulating NK cells from patients with neuroblastoma had a high frequency of NK cells activated by IL2/15, with IL2 induced CD69 upregulation notably lower. Similar trends within all three comparator groups were found for both CD56^bright^ and CD56^dim^ subsets (**Figures 2A, B**).

We next assessed IFNγ and granzyme B as key effector function molecules of NK cells. Patients with neuroblastoma failed to produce IFNγ under conditions that robustly produced IFNγ by NK cells from healthy pediatric or adult controls. In fact, the deficit in patients was even more pronounced when compared to pediatric controls, as the frequency of CD56^dim^ cells making IFNγ in healthy pediatric cells was significantly higher than their adult counterparts (**Figures 2C, D**).

In terms of cytotoxicity potential, we assessed granzyme B as a key component of the cytotoxic granule machinery. While CD56^dim^ NK cells constitutively express granzyme B, cytokines can upregulate it and indeed, induce granzyme B expression in the CD56^bright^ subset (see **Supplementary Figure 4** for representative adult histograms and **Figure 2G** for individual adult data points). IL12/15 more robustly upregulated granzyme B expression in CD56^dim^ adult NK cells compared with IL2, and this was more striking for CD56^bright^ cells. However, analysis of the other cohorts groups revealed several striking results: NK cells from healthy pediatric controls had similar IL2 upregulation of granzyme B compared to adults but appeared to have lower IL12/15 induced responses. Unexpectedly, CD56^bright^ NK cells from children with neuroblastoma dramatically upregulated granzyme B expression in response to cytokine with an average of 6-fold and 9-fold increase for IL2 and IL12/15 respectively (**Figures 2E–G**). Thus, in marked contrast to IL12/15 failure to induce IFNγ production, NK cells from patients with neuroblastoma robustly upregulated granzyme B in response to cytokine. Thus, patients retained key cytotoxic molecules required to kill cancer cells and were primed *in vivo* for further upregulation.

### NK cells from patients with neuroblastoma are primed and demonstrate enhanced metabolic engagement

**Figure 3:**
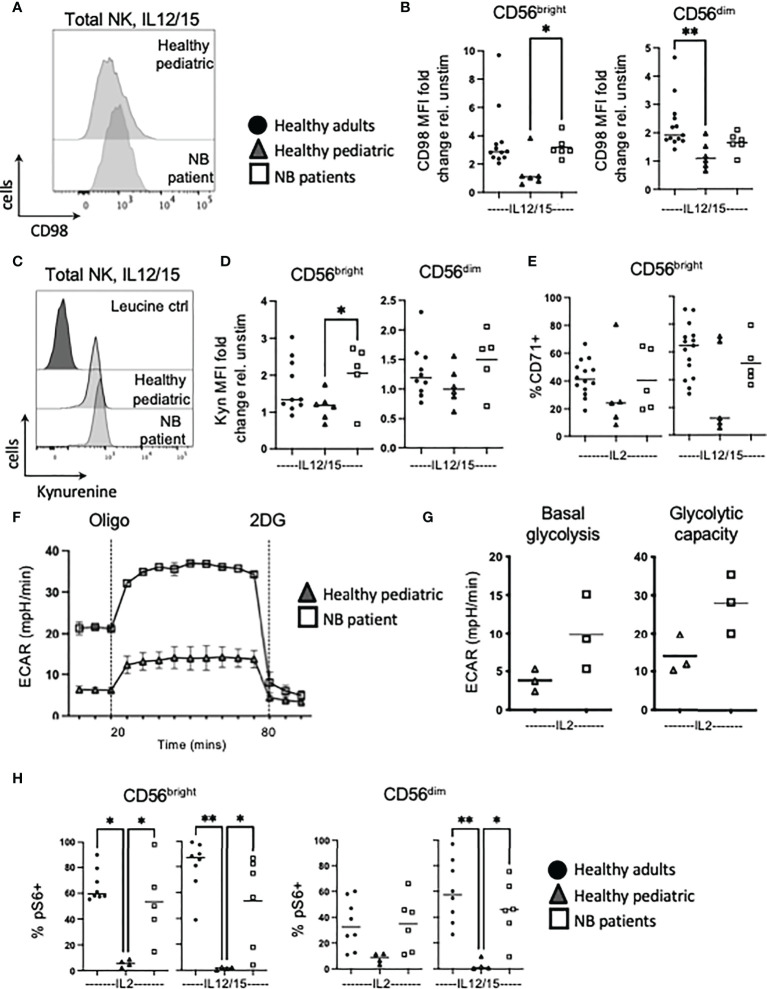
NK cells from NB patients have heightened metabolic responses and are glycolytic. PBMC were isolated from fresh blood of healthy adult donors, healthy pediatric donors and NB patients. Cells were stimulated with IL2 (500 IU/mL) or IL12 (30 ng/mL) and IL15 (100 ng/mL) at 37°C for 18 hours where indicated. **(A, B)** Cells were stained for CD98 and analyzed by flow cytometry. **(C, D)** Cells were incubated with kynurenine (200μM) and analysed by flow cytometry. Representative histograms are shown in **(C)** and dots represent individual donors and horizontal bars show the mean in **(D)**. **(E)** Cells were stained for CD71 and analyzed by flow cytometry. **(F, G)** Seahorse analysis of purified NK cells from healthy pediatric donors and NB patients was performed on the XFp extracellular flux analyzer. **(H)** Cells were stained for pS6 and analyzed by flow cytometry. Dots represent individual donors and horizontal bars show the mean, N=3-15. Samples were compared using the Two-way ANOVA test or Student’s t-test, *p<0.05, **p<0.01.

Immune cells use a range of nutrients including glucose and amino acids to fuel their metabolic activities (24–26). Given that we have previously shown that cellular metabolism is important for NK cell functions and that it is profoundly dysregulated in exhausted NK cells from metastatic breast cancer patients, we investigated for evidence of NK cell dysregulation in children with neuroblastoma. SLC7A5 (LAT1) associates with CD98 for uptake of large neutral amino acids into cells (27). In response to IL12/15 stimulation, NK cells from neuroblastoma patients significantly upregulated CD98 expression and uptake activity through SLC7A5 in CD56^bright^ cells compared to pediatric controls which unexpectedly, did not upregulate either expression or activity of this nutrient receptor (**Figures 3A–D**). A similar trend for CD71, the transferrin receptor, was seen in CD56^bright^ cells but there was variation in pediatric controls, and differences did not reach statistical significance (**Figure 3E**). Consistent throughout nutrient receptor expression and uptake experiments was that NK cells from neuroblastoma patients more resembled NK cells of healthy adults than healthy pediatric controls.

NK cells are known to upregulate glycolysis in response to cytokine and although these experiments were very difficult given the smaller blood volume allowed by our ethics, we were able to measure rates of glycolysis in response to IL2 in 3 patients with neuroblastoma and 3 pediatric controls (see **Supplementary Figure 5** for additional healthy pediatric seahorse data). The data show that while extracellular acidification rates (ECAR) were relatively low, IL2 induced higher glycolytic flux in NK cells from patients with neuroblastoma compared to healthy children (see **Figures 3F, G**). This was further supported by mTORC1 signalling data with robust phosphorylation of S6 ribosomal protein (pS6) in NK cells from children with neuroblastoma compared to pediatric controls after cytokine stimulation (**Figure 3H**). Using p4EBP1 as a second downstream readout of mTORC1 activity, similar trends were observed (see **Supplementary Figure 6**). In summary, NK cells from healthy pediatric donors only poorly upregulated nutrient uptake and glycolysis in response to cytokine. In contrast, NK cells from patients with neuroblastoma strongly engaged these responses, to a level similar to circulating NK cells of healthy adult donors. Furthermore, patient NK cells had an enhanced engagement of glycolysis in response to cytokine suggesting again that they are primed *in vivo.*

### Mitochondrial dysregulation is a feature of circulating NK cells in patients with neuroblastoma

**Figure 4:**
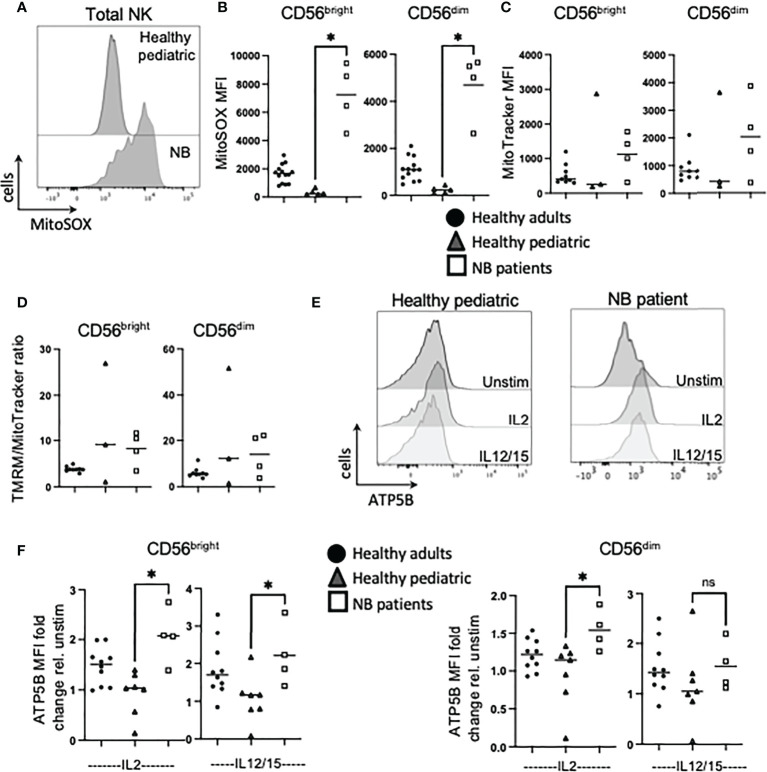
NK cells from NB patients display early signs of mitochondrial dysfunction. PBMC were isolated from fresh blood of healthy adult donors, healthy pediatric donors and NB patients. Cells were stimulated with IL2 (500 IU/mL) or IL12 (30 ng/mL) and IL15 (100 ng/mL) at 37°C for 18 hours, where indicated. **(A, B)** *Ex vivo* PBMC were stained with MitoSOX (1.5 µM) and analyzed by flow cytometry. **(C)** *Ex vivo* PBMC were stained with MitoTracker Green (100nM) and analyzed by flow cytometry. **(D)** *Ex vivo* PBMC were stained with TMRM (100nM) and MitoTracker Green (100uM), and analyzed by flow cytometry. **(E, F)** Cells were stained for ATP5B and analyzed by flow cytometry. Samples were compared using the Two-way ANOVA test. ns, not significant; * P<0.05.

**Figure 5:**
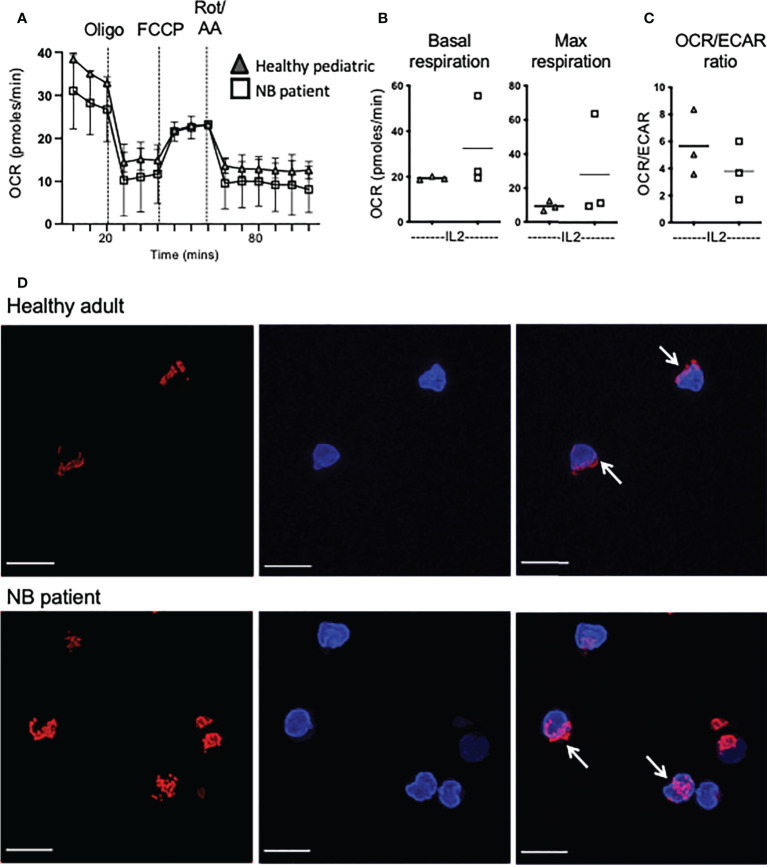
Increased dependence on glycolysis in circulating NK cells from patients with neuroblastoma. **(A–C)** Seahorse analysis of purified NK cells from healthy pediatric donors and NB patients was performed on the XFp extracellular flux analyzer. Dots represent individual donors and horizontal bars show the mean, N=3-14. Samples were compared using the Two-way ANOVA test or Student’s t-test. **(D)** Representative confocal images of purified NK cells analyzed directly *ex vivo* from a representative healthy donor and a patient with neuroblastoma (N=1) and stained with MitoSpy CMX Ros (250nM) for 30 min at 37°C and DAPI (300nM). Images shown are the Maximum Intensity projection of Z-stacks taking at 0.2μm increments. Red= Mitospy CMX Ros, Blue= DAPI. Scale bar=5μm.

Previous work showed highly dysregulated mitochondrial metabolism in circulating NK cells from adult cancer patients (19). Given the importance of mitochondria in a range of metabolic processes and pathways, including oxphos, we investigated mitochondrial health and activity in our patient cohorts. We first measured mtROS directly *ex vivo* as high levels can indicate mitochondrial stress. NK cells from healthy children had virtually no mtROS while healthy adults had consistently higher levels (**Figure 4B**). However, the children with neuroblastoma had levels of mtROS in circulating NK cells that were dramatically higher than either control cohort (**Figures 4A, B**). Mitochondrial mass levels, indicating mitochondrial abundance per cell, were more variable but data suggested that this may be higher for patients with neuroblastoma and worth further investigation (**Figure 4C**). Mitochondrial membrane potential (MMP) is required to drive ATP synthase and when MMP was normalized for mitochondrial mass, it was noted that while circulating NK cells from each adult donor had similar and consistent MMP levels, there was a lot of variability in pediatric controls and neuroblastoma patients (**Figure 4D**). Induction of metabolic machinery such as ATP5B, a component of ATP synthase complex, was induced by cytokines in adult NK cells with relatively low/no response in NK cells from healthy pediatric controls. In contrast, there was a significant increase in ATP5B protein expression in patients with neuroblastoma compared with the pediatric controls (**Figures 4E, F**). The increase in ATP5B was also more dramatic in CD56^bright^ cells. Given the increase in ATP5B, we measured oxphos (oxygen consumption rates or OCR) by extracellular metabolic flux analysis after 18h stimulation. Pediatric controls defined the basal and max respiration rates for NK cells stimulated with IL2. Two of the three neuroblastoma patients did not increase OCR levels above those of controls (**Figures 5A, B**). Combining with the glycolysis data, it can be seen that NK cells from healthy children rely primarily on oxphos but this changes in children with neuroblastoma towards a greater use of glycolysis in these NK cells (**Figure 5C**). Finally, we were able to do confocal analysis of NK cells from one patient with neuroblastoma; these NK cells had very punctate, fissed mitochondrial organization similar to that previously reported for our cohort of metastatic breast cancer patients (**Figure 5D**) (19). Overall, there are signs of emerging mitochondrial dysfunction in NK cells of patients with neuroblastoma.

### Glycolysis and not oxphos is required to fuel ADCC by NK cells

**Figure 6:**
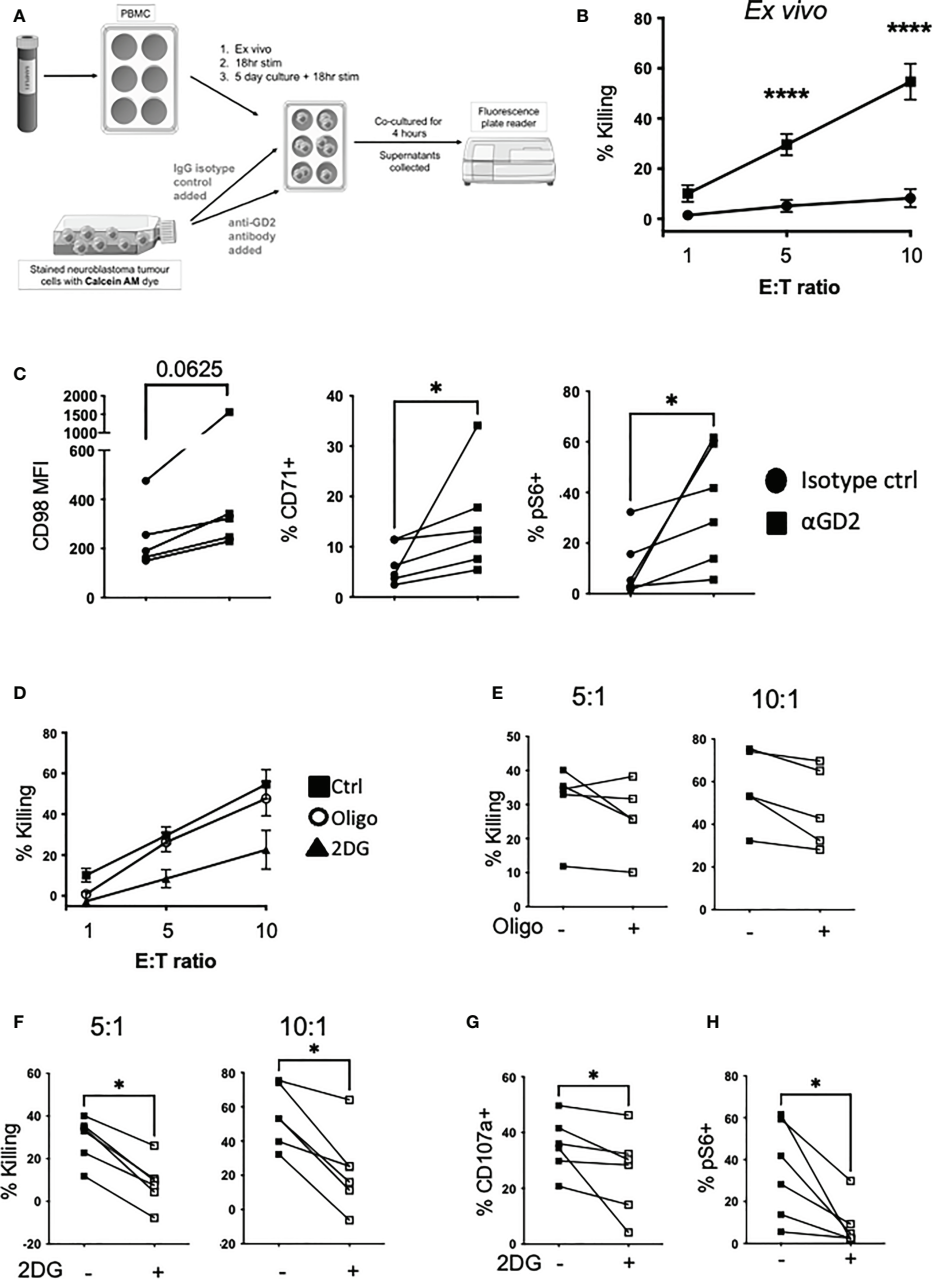
Glycolysis is required for NK cell αGD2-mediated killing of NB tumor cells. **(A)** Schematic of the setup of the ADCC assay. **(B)** Freshly isolated PBMC were cultured with Calcein AM-stained Kelly neuroblastoma cells at varying effector to target ratios (E:T) and incubated for 4 hrs at 37°C. **(C)** At the end of the assay, PBMC were stained for CD98, CD71 and pS6 analyzed by flow cytometry. **(D)** Oligomycin (40nM) and 2DG (2.5mM) was added for the 4 hour incubation. Golgi stop and anti-CD107a was added to measure degranulation. **(E, F)** Pooled data for the impact of oligomycin and 2DG on NK cells killing. **(G)** At the end of the assay, PBMC were stained for NK cell markers and pS6 and analyzed by flow cytometry. N=5-6. Samples were compared using the Two-way ANOVA test or Student’s t-test, *p<0.05, ****p<0.0001.

Given the importance of immunotherapy for high-risk neuroblastoma, we investigated if anti-GD2 engagement impacts on NK cell metabolism and the relative contribution of different metabolic pathways to support ADCC activity by NK cells. We first developed an ADCC assay in which healthy donor NK cells kill high-risk neuroblastoma tumor cells in an anti-GD2 dependent manner (**Figures 6A, B**). We explored the potential to increase NK cell killing capacity against neuroblastoma cells using a range of cytokine combinations over short and long term cultures. Under all of the conditions examined, *in vitro* culturing of NK cells prior to the ADCC assay did not increase tumor cell killing to the levels seen with *ex vivo* NK cells (approx. 40% and 60% killing for cultured and *ex vivo* NK cells respectively, see **Supplementary Figure 7**).

Engagement with anti-GD2 coated neuroblastoma cells upregulated CD98 and CD71 expression, and mTORC1 activity in NK cells compared to the isotype control (**Figure 6C**). Using oligomycin and 2-DG to inhibit oxphos and glycolytic pathways respectively in NK cells for the duration of the assay, we observed that glycolysis is the primary pathway required for NK cell killing by ADCC (**Figures 6D–F**). Glycolysis was also required for NK cell degranulation as 2DG strongly inhibited CD107a expression (**Figure 6G**). Finally, while anti-GD2 engagement induced robust activation of mTORC1 activity in NK cells, this required glycolysis and was almost fully abolished by 2DG (**Figure 6H**). Therefore, glycolysis is key to supporting both NK cell activation of mTORC1, and ADCC against neuroblastoma target cells.

## Discussion

Our results clearly show that glycolysis, and not oxphos, is required for efficient ADCC mediated by NK cells with inhibition of glycolysis impacting both ADCC and NK cell degranulation. In an era where metabolic pathways are being targeted to improve autologous and allogeneic cell therapies for cancer, these results are important as they highlight the particular relevance of discrete pathways for distinct effector functions. While targeting oxphos has been touted as a key goal for promoting mitochondrial health and longevity of therapeutic cells (28), a more balanced approach may be required where ADCC is a key component of the immunotherapy. This is particularly relevant as the selective expression of tumor associated antigens that originally facilitated development of therapeutic antibodies for lymphoma, HER2+ breast cancer and neuroblastoma, are the ideal targets for next generation CAR-T and CAR-NK therapies where both direct recognition and ADCC could synergise for optimal outcome. While cytokine stimulation did not increase ADCC above levels seen directly *ex vivo* (see **Figure 6B** and **Supplementary Figure 7**), culturing of NK cells in cytokines can alter the metabolic and functional characteristics of the cells e.g. we have previously shown that NK cell metabolism becomes mTORC1 dependent after a five days of stimulation with IL2 (29). For short term autologous cultures, protecting or indeed promoting glycolysis in NK cells might be beneficial for patients with neuroblastoma with further work required to confirm optimal conditions for longer term expanded autologous or possible adjuvant allogenic cell therapies. The impact of therapeutic antibodies directly on NK cell activity also warrants further investigation as we have some evidence that anti-GD2 engagement upregulates nutrient receptors (CD98 and CD71) and mTORC1 activity (**Figure 6C**) on NK cells.

One striking outcome of the data is that in many of the experiments, NK cells from children with neuroblastoma were more similar to those healthy normal adults that pediatric healthy controls. This highlights the importance of pediatric controls, even if not perfectly age matched, as comparisons with adult controls can lead to inappropriate conclusions e.g. cytokine upregulation of nutrient receptors CD71 and CD98 on NK cells suggests that neuroblastoma patients are ‘normal’ but comparison with the pediatric controls reveals that the these responses are actually heightened in patients. Amongst the similar responses in patients and healthy adults were *in vivo* activation (CD69 expression), mTORC1 activation and metabolic readouts including CD98 expression and ATP5B responses. It is known that inflammation is associated with aging and predisposes to a range of diseases including cancer (20, 21, 30). Our results showed that NK cells from healthy adults have a clear low level of activation/inflammation compared to healthy children, exemplified by the mtROS levels which are higher in NK cells from every adult while the healthy children have virtually no mtROS. Activation of NK cells in children with neuroblastoma presumably reflected *in vivo* systemic immune activation by the cancer as no patient had received medical interventions at time of analysis. There was a clear increase in the relative frequency of CD56^bright^ NK cells in neuroblastoma patients. Under homeostatic healthy conditions, CD56^bright^ cells are generally CD16 negative and tend to be associated with IFNγ production rather than cytotoxicity (23). While CD56^bright^ cells have the potential to differentiate towards CD56^dim^ cells (31, 32), it is also known that peripheral blood CD56^dim^ cells can upregulate CD56 expression when activated (33–35). In fact, unusual NK cell subsets have previously been reported for a number of disease states (36). The CD56^bright^ cells in our neuroblastoma patients were characterized by high CD16 expression and high levels of granzyme B and their origin requires further investigation. Interestingly, transcriptional profiling within neuroblastoma tumors recently found enriched numbers of CD56^dim^ cells in medium- and high-risk neuroblastoma, with a strong correlation of activated NK cells with survival in the overall patient cohort (20).

Patients with high-risk neuroblastoma undergo an intensive treatment regimen including chemotherapy, surgery, autologous stem cell transplant (ASCT), radiation, immunotherapy and isotretinoin treatment in that order (3, 37). By the time patients receive immunotherapy, their NK cells have been fully ablated and NK cells post-ASCT are generally immature (38) and have an altered cytotoxic phenotype in terms of subset distribution (39). Our data suggest that, prior to any intervention, NK cells in patients with neuroblastoma may be primed by the cancer itself for effective ADCC. In particular, NK cells in our patient cohort were primed to engage in glycolysis, the particular metabolic pathway required to facilitate this immune function. This could potentially be used for clinical advantage in terms of deploying immunotherapy. Rather than depending on NK cells to regenerate post ASCT, autologous circulating NK cells could be harvested soon after diagnosis for storage and/or expansion for downstream immunotherapy; this could be performed independent of autologous stem cell harvest for immune reconstitution. This relatively simple change to the current protocol has the potential for a significant clinical benefit for patients.

Mitochondria are key to many cellular processes in NK cells including energy production and are required for the synthesis of cytotoxic machinery and effector molecules (40, 41). We have previously reported that mitochondria in circulating NK cells from patients with metastatic cancer are severely dysregulated (19). They have high mitochondrial mass with fissed structures, high mtROS and are unable to engage in efficient glycolysis or oxphos in response to cytokine stimulation, as NK cells from healthy donors do. This pediatric cancer cohort revealed a more intermediate mitochondrial phenotype. Indeed, glycolytic flux was efficiently engaged in NK cells from neuroblastoma patients with unexpectedly higher basal glycolysis and glycolytic capacity in response to IL2 compared to pediatric controls. This supports the *in vivo* activation and priming of NK cells in the neuroblastoma patients in response to cancer. ATP5B levels were also induced to higher levels in the patient NK cells which suggested that these cells might also engage in high rates of oxphos. However, this was not the case and two of three patients had rates of basal and maximum respiration that were comparable to the controls. OCR/ECAR ratio analysis showed a clear shift towards a preferential use of glycolysis by patient NK cells. Thus, while NK cells in children with neuroblastoma are primed *in vivo* for enhanced glycolysis, there is evidence of emerging mitochondrial dysfunction in the cells with higher mitochondrial mass, extremely high levels of mtROS and punctate mitochondria in the one patient it was possible to analyze (see **Figures 5** and **6**); however, this dysregulation does not yet translate into impaired oxphos engagement. Thus, there may be a hierarchal loss of metabolic responses in NK cells of patients with cancer which are likely to impact on functional responses. Indeed, there was already a clear dysregulation of IFNγ production while some cytotoxicity machinery was maintained. It is interesting to speculate whether NK cell dysregulation progression patterns are disease specific. For instance, children with obesity also have dysregulated NK cells but in contrast to our cohort, they have reduced cytotoxicity, normal IFNγ production, normal mitochondrial mass and increased basal ECAR (42). Dysregulated features in common with our cohort included expansion of CD56^bright^ cells, high basal mTORC1 activity, elevated rates of glycolysis and high levels of mtROS. These data suggests that there may be early common signatures of NK cells in complex pro-inflammatory disease environments in children. This is worth further investigation in terms of specific mechanisms with a view to early, common immune protection interventions.

In summary, the data support that NK cells from patients with neuroblastoma have positive potential for autologous immunotherapy due to their maintenance of key metabolic and functional features that support glycolysis and cytotoxicity, cornerstones of current ADCC therapy. There may also be merit in harvesting autologous primed NK cells prior to cytotoxic chemotherapy with the potential to boost them genetically prior to reinfusion in an adjuvant therapeutic setting. Indeed, it is possible that autologous cellular immunotherapy is likely to be more successful and easier to achieve than in adult patients with ‘younger’ NK cells more resistant to immune exhaustion.

### Limitations of the paper

Due to rare nature of neuroblastoma (just 6 samples available from our affiliate hospital in Dublin in the last 3 years) and due to the ethical and logistical issues associated with taking blood from healthy children, our sample sizes in this study are lower than what we would ideally prefer. It will be prudent in the future to validate these finding with larger sample cohorts. Furthermore, Glutamax seahorse media was used for seahorse experiments but these findings should also be confirmed using seahorse media with normal glutamine added.

## Data availability statement

All data relevant to the study are included in the article or uploaded as supplementary information.

## Ethics statement

The Trinity College Faculty of Science, Technology, Engineering and Maths Research Ethics Committee provided ethics for analysis of healthy adult donor blood. All healthy adult donors for this study provided written consent. The ethics committee of Children’s Health Ireland at Crumlin (formerly Our Lady’s Children’s Hospital) approved the study on healthy paediatric donors and neuroblastoma patients, and parents/guardians provided written consent.

## Author contributions

KS did most of the work in terms of sample preparation, experimental procedures, data analysis, preparing figures, writing and editing of the manuscript. MB, EW, SK, and KB helped prepare and perform experiments on some of the samples. CR, SA, and AR recruited patients and pediatric healthy donors, looked after ethics and provided clinical information. CO coordinated the clinical contribution and was responsible for patient recruitment and recruitment of healthy donors, ethics applications, clinical information, reading and writing of the manuscript. CG conceived and designed the study, analysed the data, wrote the paper and revised paper. All authors contributed to the article and approved the submitted version.

## Funding

Funding for this work was provided by the National Children’s Research Centre, Crumlin, Dublin 12, Ireland (Grant ref A/18/5).

## Acknowledgments

We would like to acknowledge all the patients, their parents and carers, and of course the healthy controls who have given so generously to help this study. We also thank the phlebotomists and nursing staff for their support.

## Conflict of interest

The authors declare that the research was conducted in the absence of any commercial or financial relationships that could be construed as a potential conflict of interest.

## Publisher’s note

All claims expressed in this article are solely those of the authors and do not necessarily represent those of their affiliated organizations, or those of the publisher, the editors and the reviewers. Any product that may be evaluated in this article, or claim that may be made by its manufacturer, is not guaranteed or endorsed by the publisher.
