# Supplementary material for: Heightened metabolic responses in NK cells from patients with neuroblastoma suggests increased potential for immunotherapy
Source: Front Oncol. 2022 Oct 7;12:1004871. doi: 10.3389/fonc.2022.1004871 (PMC9585418; doi:10.3389/fonc.2022.1004871)
Supplement: Supplementary file 1 [file DataSheet_1.pdf]

Supplementary Figure 1

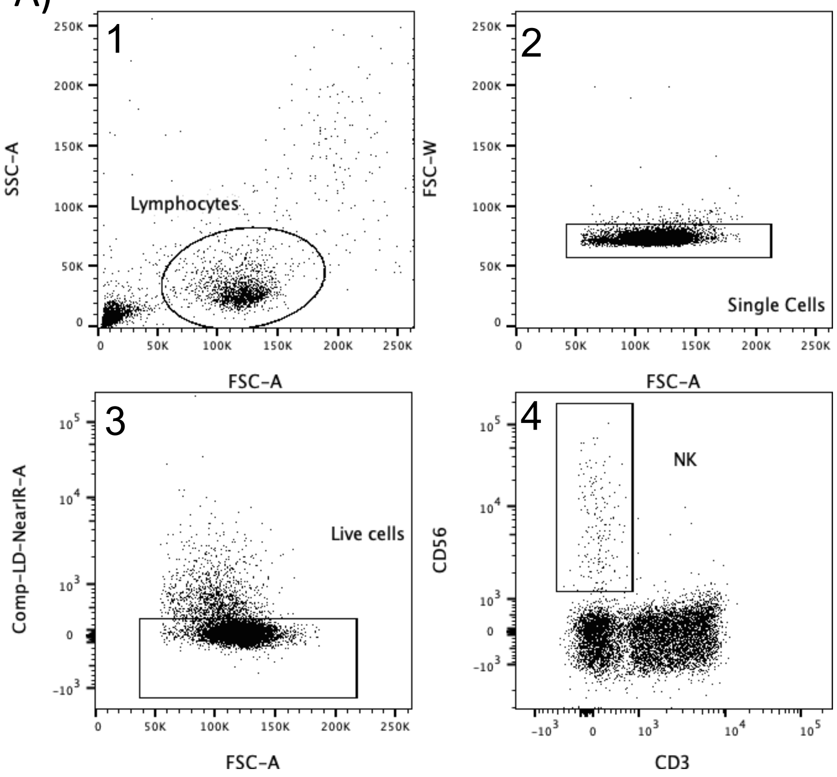

B)

Antigen	Fluorophore	Clone	Supplier
CD56	BV786	NCAM16.2	BD
CD3	Pac blue	SK7	BD
CD16	AF700	3G8	BioLegend
Granzyme B	PE-Cf594	GB11	BioLegend
IFNγ	BV605	B27	BD
CD71	APC	M-A172	BioLegend
CD69	FITC	L78	BD
CD98	PE	UM7F8	BD
pS6	Pac blue	D57.2.2E	Cell Signalling
pEBP1	PE	236B4	Cell Signalling

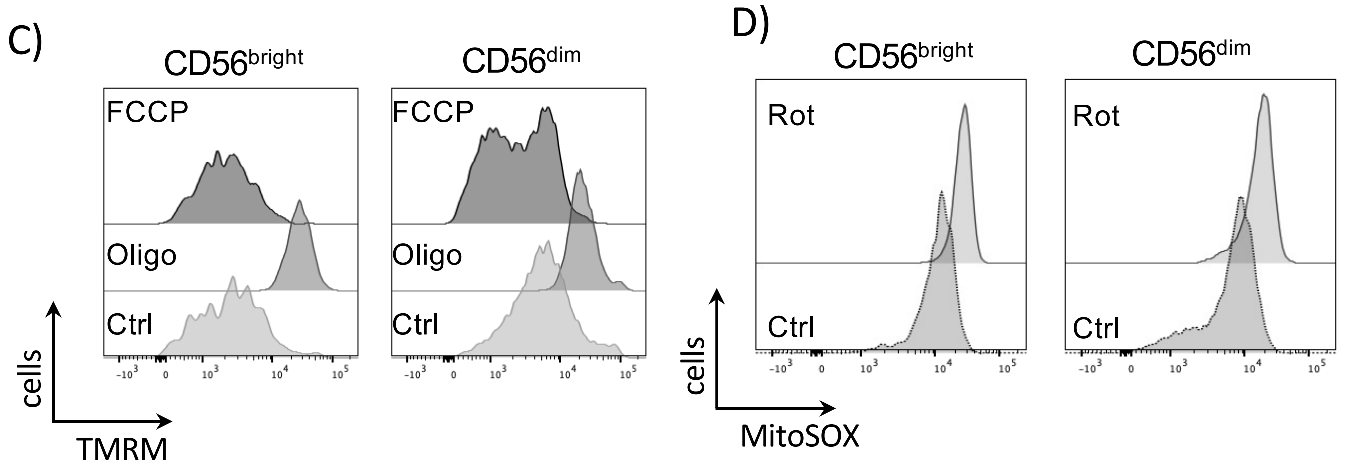

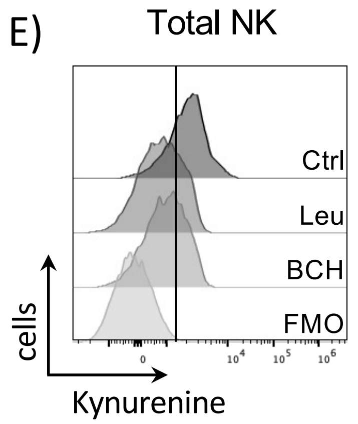

Supplementary figure 1. Gating strategy and antibodies for flow cytometry and technical controls for metabolic assays.
(A) Shows the gating strategy used to identify NK cells. (B) shows the antibodies used (C) Oligomycin (2 μM) and FCCP (2 μM) were used as positive and negative controls for the TMRM assay. (D) Rotenone (20 μM) was used as a positive control for the MitoSOX assay. (E) BCH (10mM), and inhibitor of LAT1, was used as a negative control for the kynurenine uptake assay.

Supplementary Figure 2

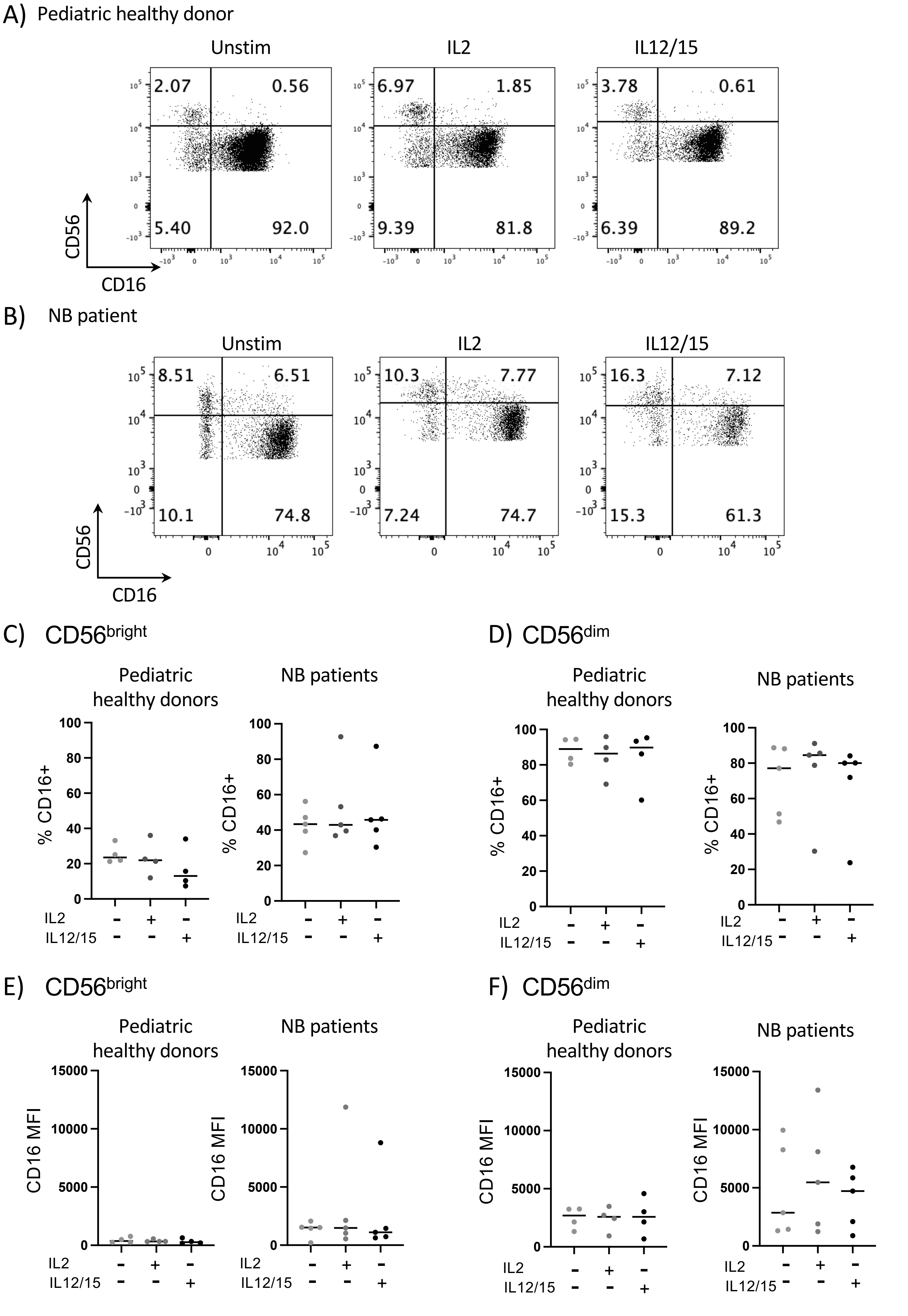

Supplementary figure 2. NK cells from NB patients express high levels of CD16.

PBMC were isolated from fresh blood of healthy adult donors, healthy pediatric donors and NB patients. Cells were stimulated with IL2 (500 IU/mL) or IL12 (30 ng/mL) and IL15 (100 ng/mL) at 37°C for 18 hours. Cells were stained for CD16 and analysed by flow cytometry. (A) Representative dot plot of CD16 staining in NK cells from a healthy paediatric donor. (B) Representative dot plot of CD16 staining in NK cells from a NB patients. (C-D) Pooled data of CD16 expression in response to cytokine stimulation. Dots represent individual donors and horizontal bars show the mean, N=4-5.

Supplementary Figure 3

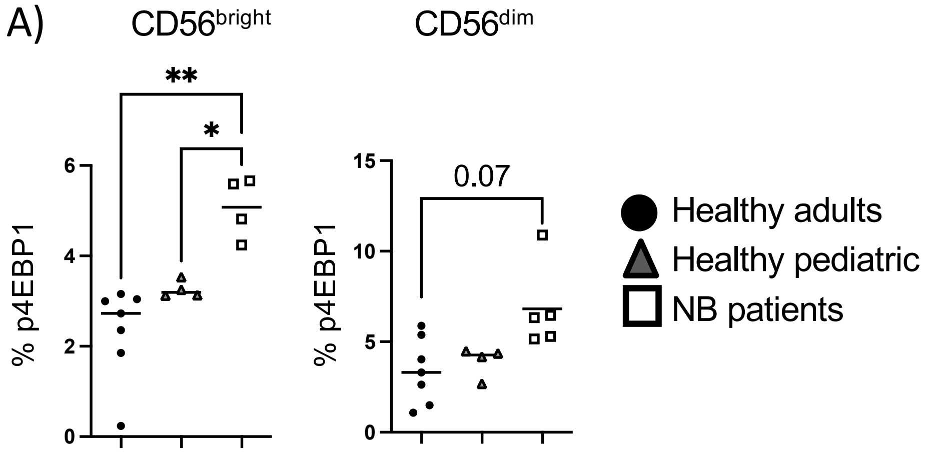

Supplementary figure 3. NK cells from NB patients have heightened mTORC1 activity.

PBMC were isolated from fresh blood of healthy adult donors, healthy pediatric donors and NB patients. PBMC were stained for p4EBP1 and analysed by flow cytometry. Dots represent individual donors and horizontal bars show the mean, N=4-7. Samples were compared using the Two-way ANOVA test, *p<0.05, **p<0.01.

Supplementary Figure 4

A) Healthy adult

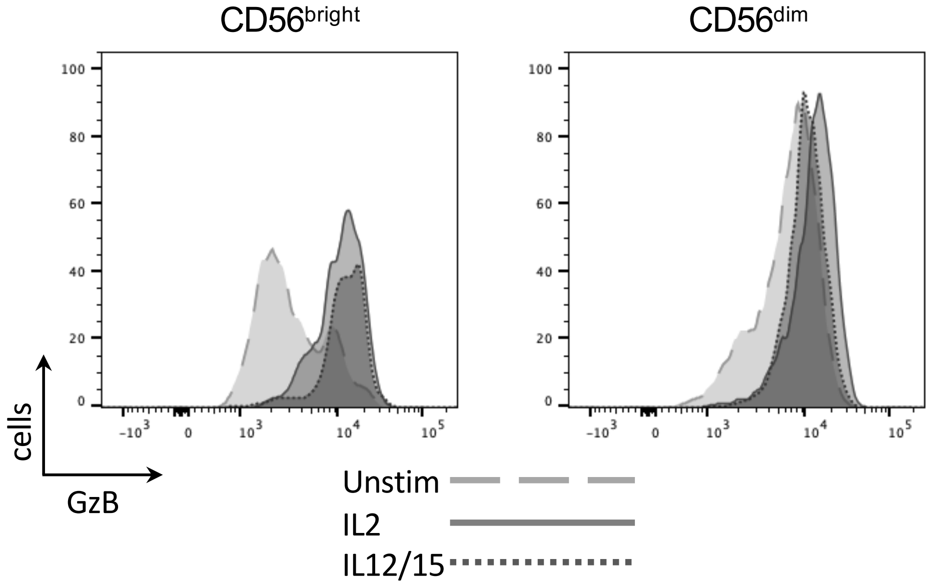

Supplementary figure 4. NK cells from healthy adults increase granzyme B expression in response to cytokine.
PBMC were isolated from fresh blood of healthy adult donors. Cells were stimulated with IL2 (500 IU/mL) or IL12 (30 ng/mL) and IL15 (100 ng/mL) at 37°C for 18 hours. Cells were stained for granzyme B (GzB) and analysed by flow cytometry.

Supplementary figure 5

Healthy pediatric donors

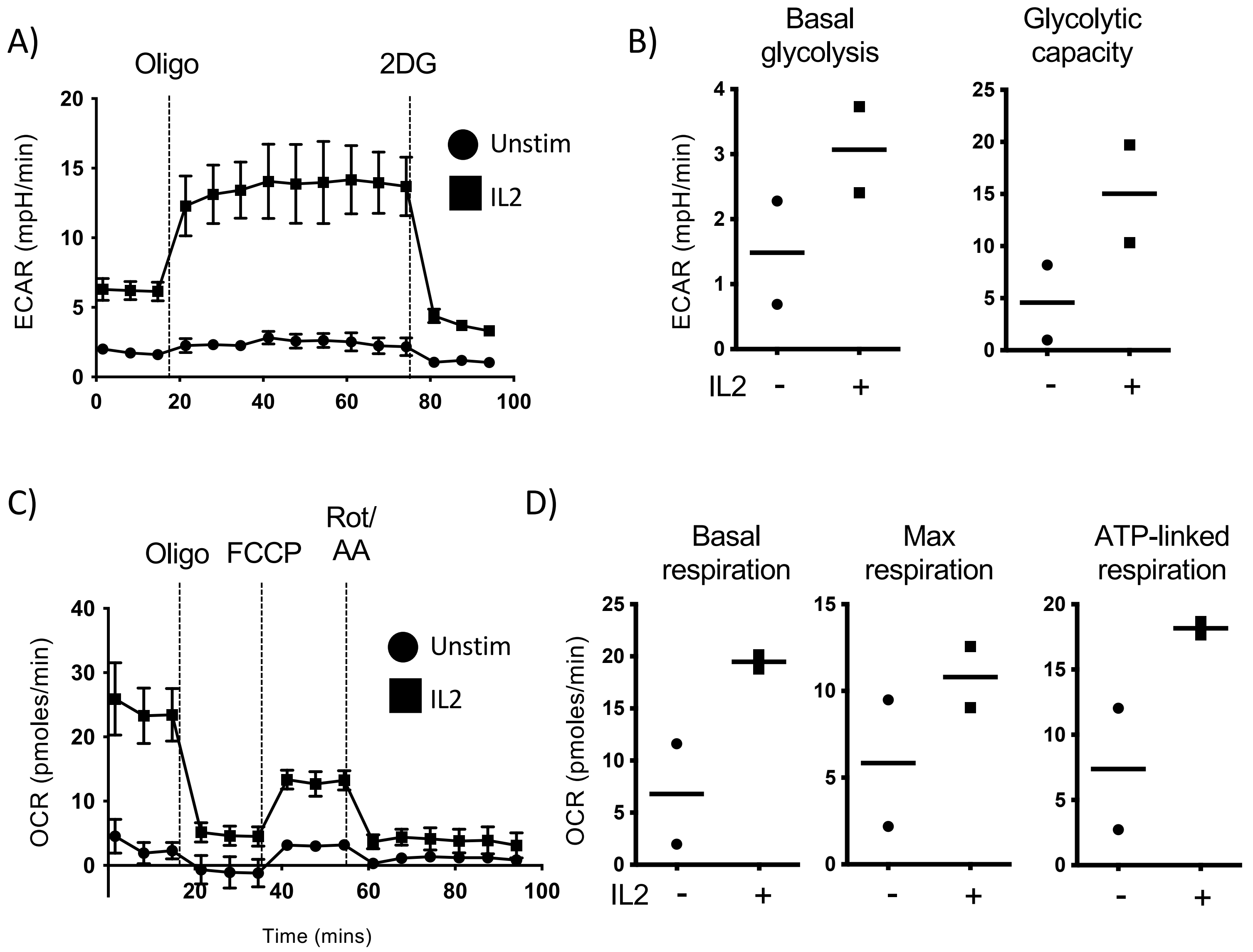

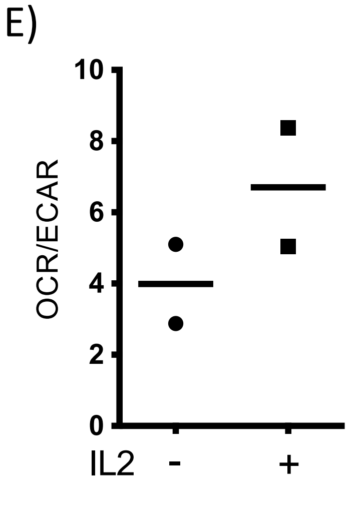

Supplementary figure 5. NK cells from healthy pediatric donors increase glycolysis and oxphos in response to IL2 stimulation.
PBMC were isolated from fresh blood of healthy pediatric donors and NK cells were purified. Cells were stimulated with IL2 (500 IU/mL) at 37°C for 18 hours. Seahorse analysis was performed on the XFp extracellular flux analyser. Dots represent individual donors and horizontal bars show the mean, N=2.

Supplementary Figure 6

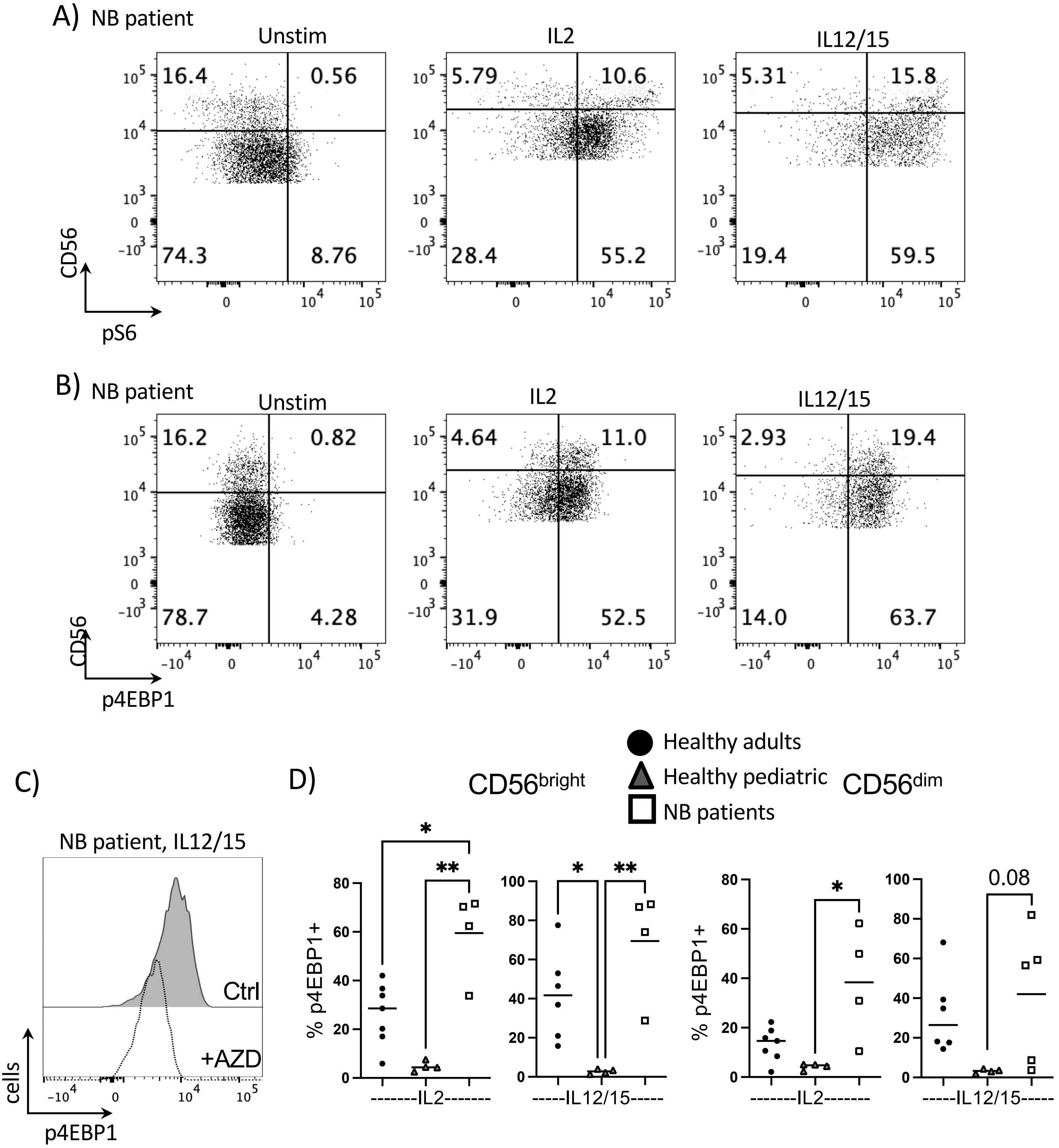

Supplementary Figure 6. NK cells from NB patients increase mTORC1 activity in response to cytokine.

PBMC were isolated from fresh blood of healthy adult donors, healthy pediatric donors and NB patients. PBMC were stained for pS6 and p4EBP1 and analysed by flow cytometry. (A) Representative dot plot of pS6 staining in NK cells from a NB patients. (B) Representative dot plot of p4EBP1 staining in NK cells from a NB patients. (C) AZD-8055 (1µM) was used as a negative control. (D) Pooled data of p4EBP1 levels in response to cytokine stimulation. Dots represent individual donors and horizontal bars show the mean, N=4-7. Samples were compared using the Two-way ANOVA test, *p<0.05, **p<0.01.

Supplementary figure 7

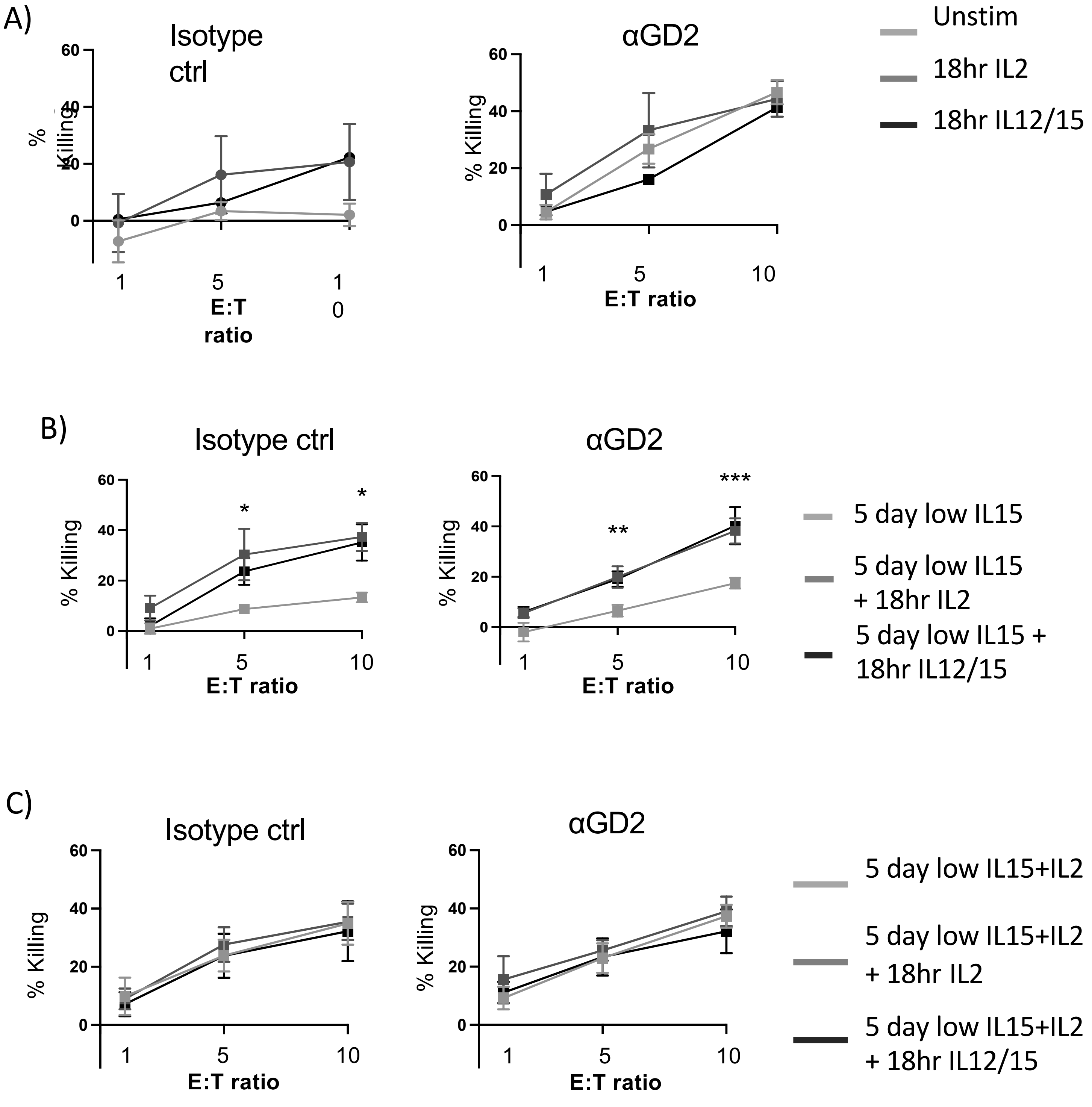

Supplementary Figure 7.
(A) Freshly isolated PBMC were stimulated with either IL2 (500IU/ml) or IL12 (30ng/ml) and IL15 (100ng/ml) and incubated for 18 hr at 37°C. (B-C) Freshly isolated PBMC were cultured for 5 days in low dose IL15 (1ng/ml, B) or low dose IL2 (2ng/ml, C). Media and cytokine were replaced on day 2 of culture. On day 5 PBMC were washed and put back at $5x10^6$ cell/ml. PBMC were left unstimulated or stimulated with either IL2 (500IU/ml) or IL12 (30ng/ml) and IL15 (100ng/ml) and incubated for 18 hr at 37°C. Kelly NB tumour cells were stained with Calcein AM dye for 30mins 37°C and rested for 1 hr. NK cells from the different cultures were cultured with Calcein AM-stained Kelly neuroblastoma cells at varying effector to target ratios (E:T) and incubated for 4 hrs at 37°C. Data points show the mean ± SEM, N=4. Samples were compared using two-way ANOVA, *p<0.05, **p<0.01, ***p<0.001.
